# Bilateral Vertebral Artery Dissections Associated With Postpartum Preeclampsia: A Case Report

**DOI:** 10.7759/cureus.98101

**Published:** 2025-11-29

**Authors:** Alexandra C Dadrat, Vittoria Costantino, Rolando J De Leon

**Affiliations:** 1 Obstetrics and Gynecology, Indiana University Health, Indianapolis, USA; 2 Physical Medicine and Rehabilitation, Florida International University, Herbert Wertheim College of Medicine, Miami, USA; 3 Obstetrics and Gynecology, Nova Southeastern University Dr. Kiran C. Patel College of Allopathic Medicine, Fort Lauderdale, USA

**Keywords:** arterial dissection in pregnancy, postpartum headache, postpartum preeclampsia, preeclampsia, vertebral artery dissection

## Abstract

Postpartum vertebral artery dissections (VADs) are infrequently reported; although data are limited, several case reports describe VAD occurring in the setting of postpartum preeclampsia. We present a 39-year-old G3P3003 (P1 term stillbirth) who developed bilateral VADs in the setting of postpartum preeclampsia and a persistent, nonfocal occipital headache.

Six days after an uncomplicated cesarean delivery, the patient developed an unrelenting occipital headache accompanied by severe-range blood pressures. She met diagnostic criteria for postpartum preeclampsia. Despite treatment with magnesium sulfate and antihypertensives, her headache persisted. On postpartum day 9, she developed new neck pain in addition to her headache. Computed tomography angiography revealed bilateral VADs. She was initiated on dual antiplatelet therapy, systemic anticoagulation, and strict blood pressure control. Her neurologic examination remained nonfocal throughout admission. Over subsequent days, her blood pressure normalized and her headache improved.

This case highlights the diagnostic challenges of evaluating postpartum headaches and underscores the importance of ongoing reassessment rather than anchoring to an initial diagnosis. It also raises the question of whether VAD precipitates hypertensive episodes or whether postpartum preeclampsia contributes to arterial injury.

## Introduction

Pregnancy and the postpartum period are characterized by substantial cardiovascular and vascular adaptations that increase arterial vulnerability. Hormonal changes, including elevated estrogen and relaxin, affect connective tissue integrity, while increased blood volume, cardiac output, and shear stress place additional strain on arterial walls. Endothelial activation and inflammation also rise in late pregnancy and early postpartum, creating a physiologic state that may predispose individuals to vascular injury [1,2]. Comprehensive reviews of hypertensive disorders of pregnancy similarly highlight these hemodynamic and vascular changes as central contributors to maternal cardiovascular risk [3].

These vascular changes become clinically significant in the development of hypertensive disorders of pregnancy, including preeclampsia. Preeclampsia results from abnormal placentation and widespread endothelial dysfunction, driven in part by excessive release of antiangiogenic factors such as soluble Flt-1 (sFlt-1), which antagonizes vascular endothelial growth factor (VEGF) and placental growth factor (PlGF) and contributes to vasoconstriction and capillary leak [2,4]. Clinically, preeclampsia is defined by new onset hypertension after 20 weeks with proteinuria or, in the absence of proteinuria, by severe features such as persistent severe range blood pressures, thrombocytopenia, renal insufficiency, transaminitis, pulmonary edema, or severe headache [5].

Another potential vascular complication associated with the postpartum state and, in some reports, with preeclampsia is vertebral artery dissection (VAD), although evidence remains limited. VAD occurs when an intimal tear allows blood to enter the arterial wall and form an intramural hematoma or false lumen that can narrow the vessel or cause thromboembolic ischemia [6,7]. Symptoms may include occipital headache, neck pain, vertigo, diplopia, dysarthria, ataxia, or focal deficits, although some patients present with nonfocal symptoms such as isolated head or neck pain [6-8]. Several case reports and observational studies suggest that pregnancy and the postpartum period are associated with an increased risk of cervical artery dissection (including carotid and vertebral), and many affected patients have hypertensive disorders of pregnancy [9-15].

## Case presentation

Our patient is a 39-year-old G3P3003 (P1 term stillbirth, P3 twin gestation) Hispanic woman who presented with severe range blood pressures and an unrelenting headache on postoperative day 6 from a repeat cesarean delivery at 37 weeks, two days of gestation. The pregnancy was uncomplicated, and she had been discharged home normotensive.

On postoperative day 5, she developed a new occipital headache that she attributed to fatigue. By postoperative day 6, the headache had progressed to constant severe pain unresponsive to acetaminophen or ibuprofen. She described a pressure and throbbing sensation in the occipital region and denied visual changes, photophobia, nausea, vomiting, chest pain, shortness of breath, focal weakness, numbness, speech difficulty, or imbalance.

Blood pressure on presentation reached 179/112 mm Hg. She appeared uncomfortable but was alert and oriented with normal cardiopulmonary and abdominal findings. Her neurologic examination was nonfocal. Laboratory values were as follows: hemoglobin 12.4 g/dL; platelets 234 × 10^9^/L; creatinine 0.63 mg/dL; aspartate aminotransferase (AST) 23 U/L; and alanine aminotransferase (ALT) 19 U/L. D-dimer, fibrin monomer, lactate dehydrogenase (LDH), and uric acid were not obtained.

Given her severe postpartum headache, the differential diagnosis included migraine, tension-type headache, post-dural puncture headache, preeclampsia with severe features, posterior reversible encephalopathy syndrome, cerebral venous sinus thrombosis, ischemic stroke, and cervical artery dissection.

A diagnosis of postpartum preeclampsia with severe features was made based on severe-range blood pressures and persistent headache unresponsive to treatment. Proteinuria testing was not obtained because ongoing lochia interferes with accuracy and would not have changed management [5]. She received intravenous magnesium sulfate for seizure prophylaxis and guideline-based antihypertensive therapy with nifedipine.

**Figure 1:**
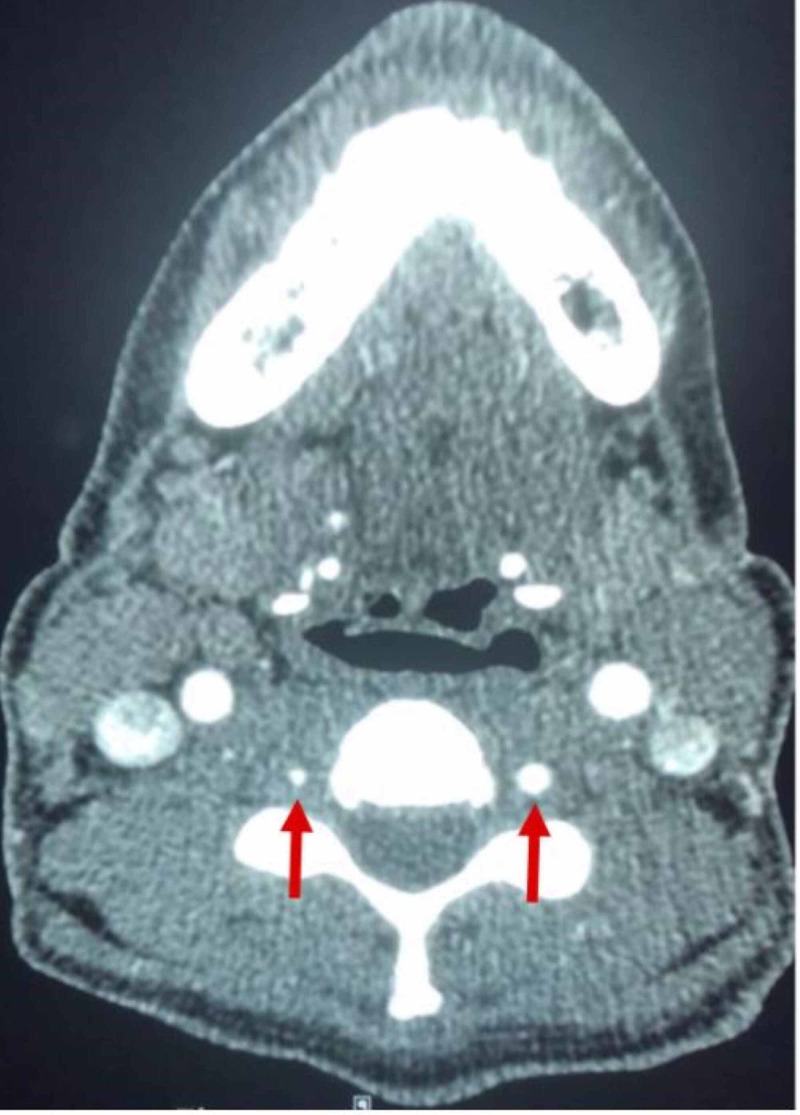
Vertebral Artery Narrowing Axial CT angiography (CTA) showing a narrowing of the vertebral artery lumen on the left than on the right (arrows).

**Figure 2:**
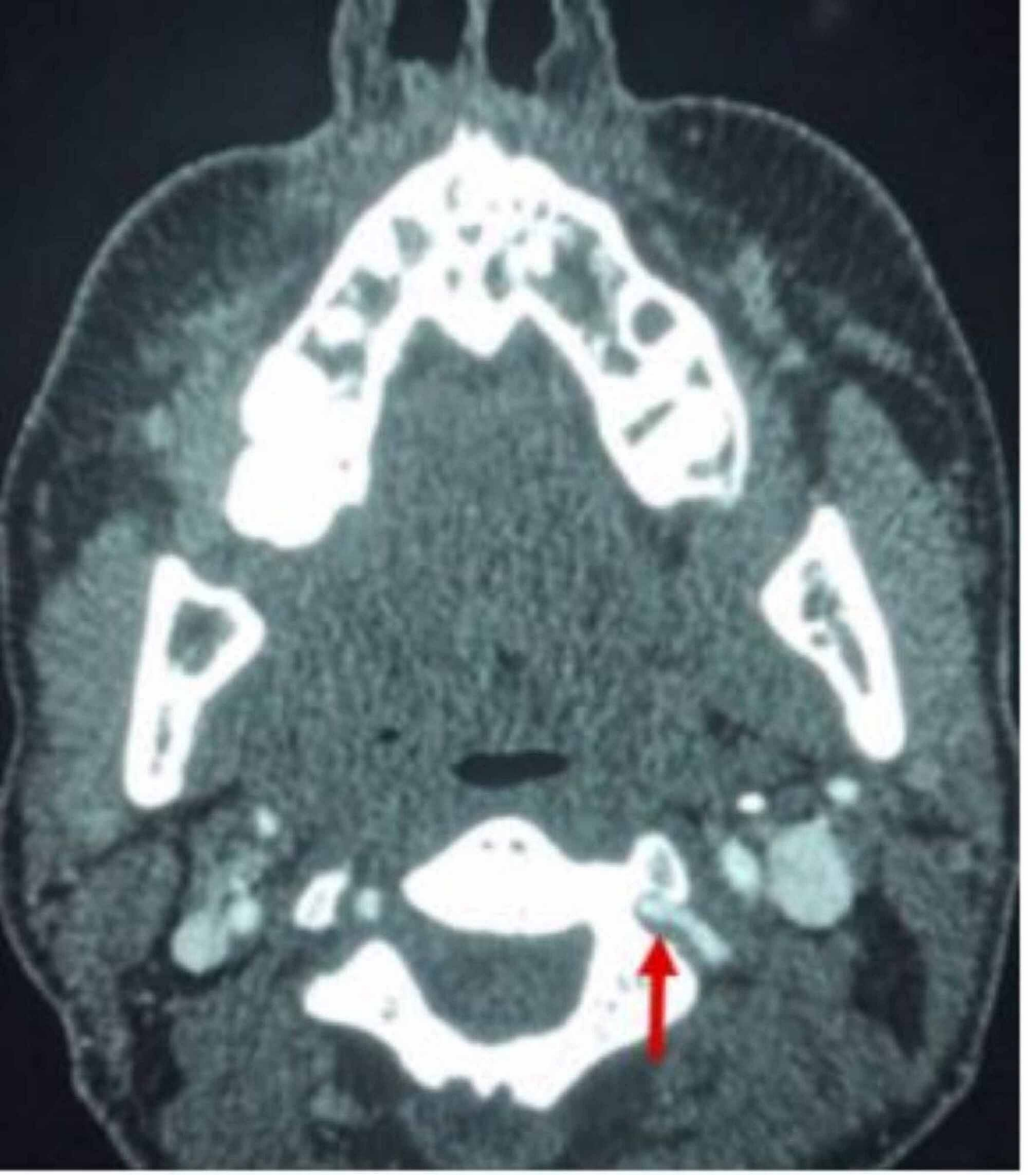
Left Vertebral Artery Intimal Flap Axial CT angiography (CTA) showing a patent intimal flap with possible thrombosis in the left vertebral artery (arrow).

On postoperative day 9, she reported new bilateral neck pain in addition to the occipital headache. She remained without focal neurologic deficits. Given this symptom evolution, computerized tomography angiography was performed and demonstrated segmental narrowing of both vertebral arteries, more pronounced on the left (Figure 1). Neurointerventional evaluation confirmed subtle dissection flaps bilaterally (Figure 2). Magnetic resonance imaging showed no infarction, hemorrhage, or posterior reversible encephalopathy syndrome, and magnetic resonance venography was normal.

Neurology recommended medical management. She received intravenous heparin and was then transitioned to dual antiplatelet therapy with aspirin and clopidogrel per neurointerventional recommendations. Nifedipine was started for blood pressure control. A cervical collar was used for stabilization. As blood pressure improved, her pain resolved. She remained neurologically intact and was discharged with outpatient follow-up. At six weeks postpartum, she reported complete resolution of symptoms.

## Discussion

This case highlights the diagnostic complexity of postpartum headache, particularly in patients with risk factors for hypertensive disorders of pregnancy. Although postpartum headache is often benign, it can signal serious pathology such as VAD, cerebral venous sinus thrombosis, posterior reversible encephalopathy syndrome, or stroke. The overlapping presentation of VAD and preeclampsia can obscure timely diagnosis, as both may manifest with severe headache, elevated blood pressure, and a nonfocal neurologic examination. Additionally, endothelial dysfunction and vasospasm associated with preeclampsia, including elevated sFlt-1 levels, may contribute to arterial susceptibility, while pain from evolving dissection may provoke reactive hypertension [16].

Posterior reversible encephalopathy syndrome was considered, given the patient’s symptoms and postpartum status, but the absence of visual disturbances, seizures, or imaging abnormalities made this diagnosis unlikely. Its inclusion in the differential emphasizes the importance of prompt neuroimaging when the clinical trajectory deviates from an expected postpartum course [17].

The key diagnostic turning point in this case was the development of new bilateral neck pain, a symptom atypical for preeclampsia but characteristic of cervical artery dissection. Symptom evolution should prompt neurovascular imaging even when neurologic examinations remain normal. Early recognition is essential to reduce the risk of ischemic complications and to guide appropriate management.

Compared with previously reported postpartum or pregnancy-associated vertebral or cervical artery dissections, our case diverges in two important respects. First, in the majority of prior series, the dissection is unilateral; for example, Shanmugalingam et al. reported four patients (two antenatal, two postpartum) each with a single vertebral artery lesion [11], and Gasecki et al. described several postpartum dissections but likewise single-vessel involvement [9]. In contrast, our patient had bilateral VADs, which, to our knowledge, are exceptionally rare in this context. Second, many published cases are accompanied by focal neurologic deficits or infarction at presentation; our patient remained neurologically intact throughout and exhibited nonfocal symptoms (headache and neck pain only) despite bilateral arterial involvement and imaging-confirmed dissections. These differences emphasize the broadening clinical spectrum of postpartum VAD and support the need for vigilance even in the absence of focal neurologic signs.

Management of VAD typically includes antiplatelet therapy or anticoagulation, with endovascular intervention reserved for specific scenarios such as hemodynamic compromise, progressive luminal narrowing, pseudoaneurysm formation, or high-risk vascular anatomy [6,7]. Most patients with preserved distal flow and nonfocal neurologic examinations have favorable outcomes with medical therapy alone. In this case, the patient’s stable neurologic status, absence of infarction, and maintained antegrade flow supported conservative management, which aligns with current evidence.

Limitations of this case include the lack of proteinuria quantification due to interference from lochia, incomplete coagulation testing, and uncertainty regarding the precise timing of arterial injury. As with all case reports, causality cannot be inferred from temporal association alone, and further study is needed to clarify the relationship between hypertensive disorders of pregnancy and cervical artery dissections.

## Conclusions

This case underscores the importance of maintaining a broad differential diagnosis for postpartum headache and reassessing the initial diagnosis when symptoms evolve. Although preeclampsia was an appropriate early consideration, the development of neck pain prompted neurovascular imaging that identified bilateral VADs. Cervical artery dissection should remain a consideration even when neurologic examinations are normal. As a single case report, this observation cannot establish causality between hypertensive disorders of pregnancy and arterial dissections, and further study is needed to guide optimal evaluation and management.
